# Vienna Letter

**Published:** 1881-01

**Authors:** W. T. Belfield

**Affiliations:** Vienna


### Foreign Correspondence.

ARTICLE X.

VIENNA, November 20, 1880.

EDITORS MEDICAL JOURNAL AND EXAMINER:

The winter session opened October 11, with a large attendance, including a delegation of some 150 Americans. New York and Boston furnish, perhaps, one-half of our countrymen here, the Western cities being rather scantily represented. Chicago has at present four representatives here; St. Louis and Cincinnati each three.

The university has recently sustained a severe loss, as you have doubtless learned, in the death of Prof. Hebra, and has this week been called upon to mourn the loss of the senior surgeon, Dumreicher, who enjoyed a very high European renown, though in America not so famous as his colleague, Billroth. Hebra has been succeeded by his son-in-law, Kaposi, for many years his hospital assistant. Dumreicher's successor will probably be Czerny, of Heidelberg.

The teaching in obstetrics here offers several points of interest. They esteem very highly, and practice very skillfully, the *external* examination of the pregnant woman, a proceeding by which the position and presentation of the child can be in almost all cases accurately determined without vaginal examination, in some instances even when the latter proceeding gives negative results. The advantages of such a measure, more especially in private practice, are obvious. They are very successful, too, in avoiding serious ruptures of the perineum. For this purpose they oppose the uterine contractions as soon as the perineum is somewhat distended, pressing the head not only upwards against the perineum, but also backwards toward the promontory. If rupture still threatens they make epiphyseotomy, cutting one or both labia slightly, just below the venous plexus. After the expulsion of the child the hand is never, under ordinary circumstances, introduced into the vagina; the placenta is expelled by Crédé's method and carefully examined as to its integrity; the parturient canal, uterus included, is carefully and thoroughly washed out with a two per cent. carbolic acid solution applied by means of a fountain syringe. The uterus is compressed and kneaded daily for three days after delivery, each compression being followed by an irrigation. To these measures is credited the fact that among the ten thousand annual deliveries in the hospital, many of them attended by men who are at the same time dissecting, puerperal fever is a comparative rarity.

The application of the forceps is here considered advisable whenever the head has for three hours failed to advance. Simpson's forceps is almost invariably used. Braun recently exhibited and discussed the very elaborate new forceps of Tarnier, deeming it a useful instrument in special cases only.

Braun advocates very strongly the extirpation of the pregnant uterus with ovaries as a substitute for the Cæsarean section, not simply because of the subsequent immunity from similar operations conferred upon the patient, but because the operation is, in his opinion, less dangerous than the Cæsarean. He shows a record of six cases with four recoveries. His colleagues, however, both in and out of Vienna, are by no means so sanguine; indeed the collective statistics scarcely give the operation any claim to superiority over the old one, the results in Italy, where the measure originated, having been especially unfavorable. Braun maintains his opinion, however, and hopes to repeat the operation soon on a woman with deformed pelvis—conjugate diameter six centimeters—now in the hospital.

The point is really one of some importance here in Austria, where rachitis is so prevalent among children, and hence, deformed pelvis, so numerous among adults.

In gynæcology I have seen nothing especially new. They tacitly admit here that America leads the world in this branch, and speak with generous admiration of Sims and Bozeman, from whom they confess to have learned several things about operations on the cervix, and on vaginal fistulæ.

Stricker is out this year with a series of new observations upon points both old and new. Among other things he fires, to use his own words, "the last dart required to demolish" Cohnheim's emigration theory as to the origin of pus. By means of artificial irritation, Stricker induces suppuration on the center of a frog's cornea, a tissue which, as is well known, contains no blood-vessels in the normal state, and in which none are developed until after the appearance of pus around the focus of irritation. In this tissue he demonstrates to his class the formation of pus through the swelling and subdivision of the corneal cells, the intercellular substance at the same time disappearing. No emigration of corpuscles to the inflamed point can be discovered. Stricker insists that Cohnheim, while demonstrating indisputably the emigration of the white corpuscles, quietly assumed that these corpuscles constituted pus.

His chief efforts however, have been devoted to the elucidation of several points in the pathology of the nervous system, employing for this purpose, in addition to other lower animals, several apes. His results I shall hope to communicate to the JOURNAL in my next.

W. T. BELFIELD.

---

BINDING ADVERTISEMENTS IN MEDICAL BOOKS.—Blending books and catalogues is never agreeable, but when it comes to putting in about as much catalogue as there is book it is time that something were said against it in self-defense. No less than *sixty* pages of a book-list appear in the recent reprint of Savage's *Female Pelvic Organs*, published in Wood's Standard Series for 1880. What otherwise must have proved in every way an attractive volume is completely spoiled in its appearance. As a specimen of book-making, aside from its well-executed plates, it is decidedly the most execrable that has confronted us for many months.—*Medical Library Journal.*

